# The outcome of intraoral onlay block bone grafts on alveolar ridge augmentations: A systematic review

**DOI:** 10.4317/medoral.20194

**Published:** 2015-02-07

**Authors:** Amparo Aloy-Prósper, David Peñarrocha-Oltra, Maria A. Peñarrocha-Diago, Miguel Peñarrocha-Diago

**Affiliations:** 1 Master of Oral Surgery and Implant Dentistry. Valencia University Medical and Dental School, University of Valencia, Spain; 2 Full Professor of Oral Surgery. Master of Oral Surgery and Implant Dentistry. Valencia University Medical and Dental School, University of Valencia, Spain; 3 Chairman of Oral Surgery. Director of the Master Program in Oral Surgery and Implant Dentistry. Valencia University Medical and Dental School, University of Valencia, Spain

## Abstract

Aim: The purpose of this study was to systematically review clinical studies examining the survival and success rates of implants placed with intraoral onlay autogenous bone grafts to answer the following question: do ridge augmentations procedures with intraoral onlay block bone grafts in conjunction with or prior to implant placement influence implant outcome when compared with a control group (guided bone regeneration, alveolar distraction, native bone or short dental implants.)? Material and Method: An electronic data banks and hand searching were used to find relevant articles on vertical and lateral augmentation procedures performed with intraoral onlay block bone grafts for dental implant therapy published up to October 2013. Publications in English, on human subjects, with a controlled study design –involving at least one group with defects treated with intraoral onlay block bone grafts, more than five patients and a minimum follow-up of 12 months after prosthetic loading were included. Two reviewers extracted the data. Results: A total of 6 studies met the inclusion criteria: 4 studies on horizontal augmentation and 2 studies on vertical augmentation. Intraoperative complications were not reported. Most common postsurgical complications included mainly mucosal dehiscences (4 studies), bone graft or membrane exposures (3 studies), complete failures of block grafts (2 studies) and neurosensory alterations (4 studies). For lateral augmentation procedures, implant survival rates ranged from 96.9% to 100%, while for vertical augmentation they ranged from 89.5% to 100%. None article studied the soft tissues healing. Conclusions: Survival and success rates of implants placed in horizontally and vertically resorbed edentulous ridges reconstructed with block bone grafts are similar to those of implants placed in native bone, in distracted sites or with guided bone regeneration. More surgical challenges and morbidity arise from vertical augmentations, thus short implants may be a feasible option.

** Key words:**Alveolar ridge augmentation, intraoral bone grafts, onlay grafts, block grafts, dental implants.

## Introduction

Localized or generalized bone defects of the alveolar ridge, due to atrophy, periodontal disease and trauma sequelae, may provide insufficient bone volume or unfavorable vertical, transverse, and sagittal inter arch relationship, which may render implant placement impossible or incorrect from a functional and esthetic viewpoint (1). A variety of surgical procedures have been proposed to augment the local bone volume of deficient sites, such as: autogenous bone grafts, guided bone regeneration and alveolar distraction osteogenesis (2,3).

However, despite a relevant number of publications reporting favorable results with these different surgical procedures, considerable controversy still exists as far as the choice of the most reliable technique is concerned; this is frequently due to the lack of comparative studies (1). Six systematic literature reviews involving implants placed in lateral or vertical atrophic ridges regenerated with intraoral block bone grafts have found evidence of bone gain and high implant success rates (1,3-7). Nevertheless this technique is associated with a relevant morbidity, and the resorption of a significant part of the graft or its exposure are two of the most frequently reported complications (8,9). This is especially important in vertical augmentation procedures because the forces exerted on the graft when the soft tissue envelope expands vertically may lead to major resorption (10).

The aim of this study was to systematically review the following question: In patients with localized alveolar ridge defects, how do clinical and radiographic outcomes obtained with augmentation with intraoral autogenous block bone grafts compare with those of other techniques (guided bone regeneration, native bone, distraction osteogenesis, or short implants)? This was done by assessing the complications related to the augmentation procedure, graft success, implant survival, implant success, and radiographic peri-implant marginal bone loss.

## Material and Methods

This systematic review complies with the PRISMA statement (Preferred Reporting Items for Systematic reviews and Meta-Analyses) (11).

- Inclusion criteria 

Inclusion and exclusion criteria were established before carrying out the literature search. Inclusion criteria were as follows: (1) publications in English; (2) studies on human subjects; (3) a controlled study design –involving at least one test group with patients treated with intraoral onlay block bone grafts. If both groups compared block bone grafts, control and test group were considered according to the criteria of each individual study; (4) patients had to be rehabilitated with dental implant therapy; (5) studies involving more than five patients in each group; (6) studies had to specify the survival rate (and success rate when available) of implants; and (7) a minimum follow-up of 12 months after prosthetic loading.

Exclusion criteria were: case reports, reviews, or technical notes; studies on sinus bone grafting, studies only providing histological data or volumetric measurements (i.e. bone gain and resorption but no data about dental implants); patients affected by bone defects following ablation due to tumors or osteoradionecrosis; bone defects related to congenital malformations (such as cleft lip and palate or major craniofacial malformations), as the initial clinical situation is very different and not comparable to defects following atrophy, periodontal disease, or trauma; studies including both lateral and vertical augmentation procedures but which did not separate dental implant data according to augmentation procedure and studies with missing data.

No restrictions were placed on the year of publication. Authors were contacted for clarification of missing information when necessary.

- Outcome measures and follow-up period

The survival rate was presented (when possible) as a cumulative survival percentage rate indicating that a certain percentage of implants were still present in the mouth at the end of the observation period. Any other definitions of implant survival, as described in individual studies, were also considered. Due to the lack of consensus regarding a set of universally accepted success criteria, all definitions of implant success were considered according to the criteria of each individual study. Mean marginal peri-implant bone loss was collected (when possible). All intra or postoperative complications reported in the studies were collected.

- Initial literature search 

The Pubmed (MEDLINE) database of the United States National Library of Medicine was used for a literature search of articles published until October 2013. The following terms were used in different combinations: ‘bone grafts,’ AND ‘dental implants’ AND ‘humans’ AND ‘augmentation’, NOT ‘sinus’. This search was combined with the following search terms: ‘simultaneous’, ‘delayed’, ‘intraoral’, ‘onlay’, ‘block’, ‘horizontal’, ‘vertical’. Duplicates were removed from the search.

The search was completed by a review of the references given in each of the studies found in order to identify any additional studies that the initial search might have missed. In addition, a manual search in the private library of MP which included the following journals: British Journal of Oral and Maxillofacial Surgery, Clinical Oral Implants Research, Clinical Implant Dentistry and Related Research, International Journal of Oral and Maxillofacial Implants, International Journal of Oral and Maxillofacial Surgery, Oral Surgery Oral Medicine Oral Pathology Oral Radiology and Endodontology, Journal of Oral and Maxillofacial Surgery, and Medicina Oral Patología Oral y Cirugía Bucal.

- Searching for relevant studies.

**Figure 1:**
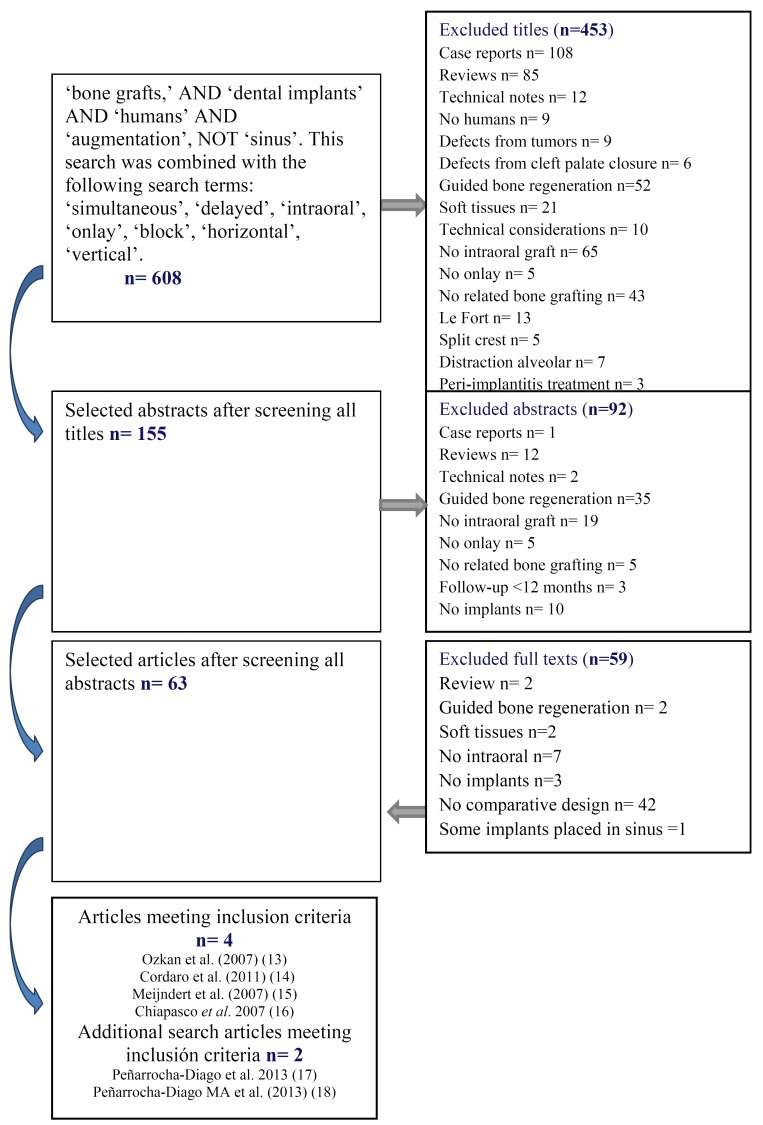
Flow diagram showing study selection for the review.

The comprehensive nature of the search methodology produced a large volume of published studies related to the topic. A three-stage screening process was then performed independently in duplicate to maximize the reliability of the extracted data (Fig. 1). Independent duplicate data extraction was carried out by two reviewers (AAP and DPO), using a predetermined data extraction form (at the third screening stage). During each stage, all disagreements were resolved by discussion and if necessary, a third reviewer was consulted (MP). The first stage screened titles in order to eliminate irrelevant publications. The second stage filtered abstracts on the basis of the number of patients, the type of graft, the intervention and the outcome characteristics. The third stage consisted of a full reading of each text using a predetermined data extraction form to confirm study eligibility on the basis of the predetermined inclusion and exclusion criteria (Fig. 1). The level of agreement regarding inclusion of potential studies was calculated by k-statistics for the second and third stage of screening.

- Quality and risk of bias assessment

Two reviewers independently and in duplicate evaluated the quality of the included studies as part of the data extraction process. Four main quality criteria were examined: (1) concealment of allocation; (2) assessor blinding; (3) patient blinding; and (4) compliance with follow-up (withdrawals in the case of a clear explanation for removals and dropouts in each treatment group). The publications were grouped into the following categories: (A) low risk of bias (possible bias not seriously affecting the results) if all the criteria were met; (B) high risk of bias (possible bias, seriously weakening the reliability of the results) if one or more criteria were not met.

- Data synthesis and analysis 

Evidence tables were created with the study data. An initial descriptive analysis (summary) was performed to determine the quantity of data, at the same time assessing variations in study characteristics. The following information was collected from the publications: type of study, type of procedure, number of treated patients (gender and age), number of implants, donor site of the grafts, delayed or simultaneous implant placement, follow-up (months), implant survival and success rates, mean marginal bone loss (mm), number and type of intra/postoperative complications.

## Results

- Study selection and description

The electronic and hand searches yielded a total of 608 articles. 453 studies were excluded after screening the titles and 92 after reading the abstracts. Overall, 63 full articles were retrieved for more detailed evaluation but only 4 fulfilled the inclusion criteria for data extraction (Fig. 1). The k value for inter-reviewer agreement for study inclusion was 0.87 (titles and abstracts) and 1.0 (full-texts) indicating ‘‘good’’ and ‘‘complete’’ agreement, respectively, between reviewers according to criteria put forward by Landis & Koch (12). Based on an additional search, 2 articles were included in this review. The results are presented separately for the horizontal and the vertical augmentation procedures.

- Horizontal augmentation

Patient and intervention characteristics

Four studies were identified involving a total of 167 patients and 254 implants. A total of 160 patients with 216 dental implants received lateral ridge augmentation with intraoral block bone grafts: 38 were placed simultaneously and 216 delayed to the bone grafting procedure (see  Table 1 for further details).

**Table 1:**
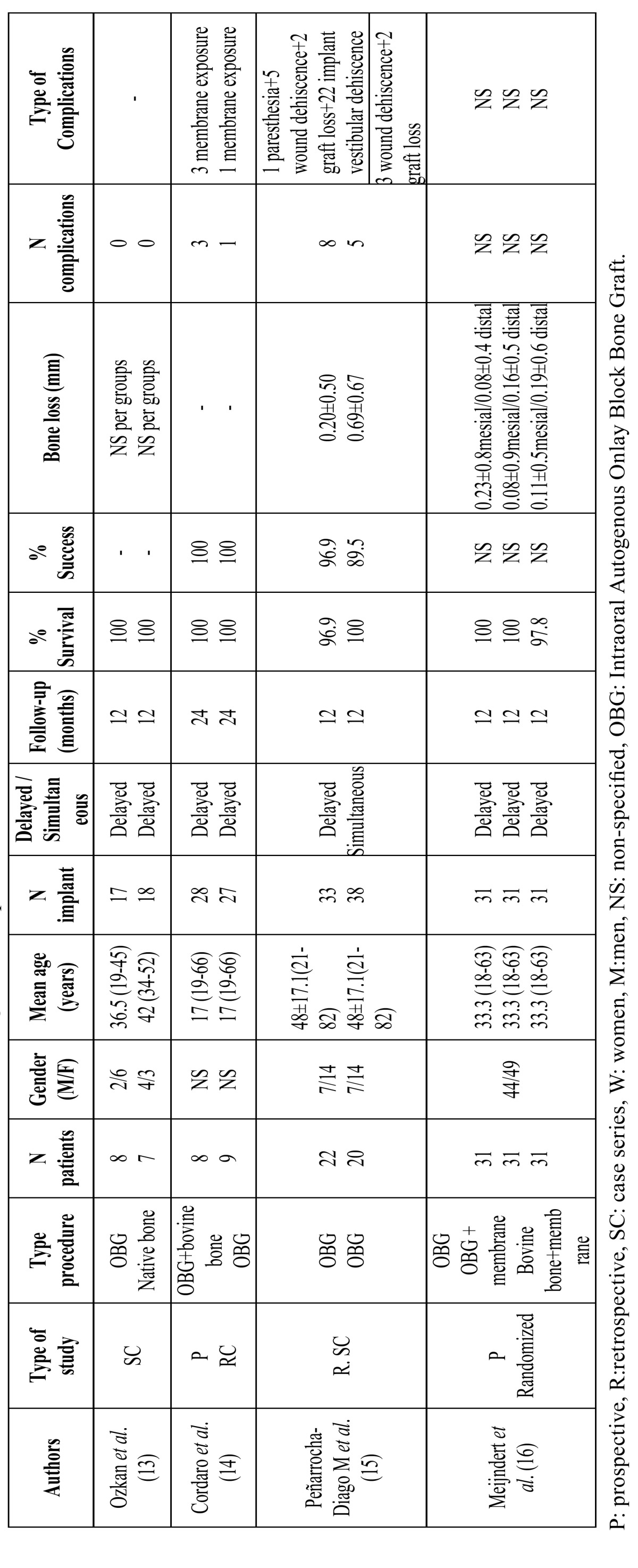
Studies included in the review on horizontal augmentation procedures.

Outcomes

Regarding surgical complications, postoperative morbidity was related to soft tissue management. The most frequent post surgical complications included mucosal dehiscences with or without exposure of the grafts or membrane (4 cases of membrane exposure and 17 graft exposures) (13,14). In most cases, membrane exposure reepithelized with no further trouble after treatment with clorhexidine three times per day for ten days, even after the removal of a part of the graft with a bur (14). If exposure increased, the complete removal of the graft was required (15). Complete failure of the block bone grafts was reported by Peñarrocha-Diago *et al*. (15). The same authors (15) also reported temporary neural disturbances involving branches of the inferior alveolar nerve; especially when grafts were from the mandibular ramus. The majority of the studies harvested the bone grafts from chin and ramus or retromolar area. No information regarding complications was found in the remaining studies (13,16). None article studied the soft tissues healing or esthetic outcomes.

Implant survival for delayed implant placement varied from 96.9% at one year post-loading (15) to 100% (13,14) with a mean of follow-up period of 12-24 months. For simultaneous implants placement (only 1 study) reported 100% at 12-months of follow-up (15). With regard to precise criteria-based definitions of implant success, three studies specified the criteria applied for evaluating implant success. Peñarrocha-Diago *et al*. (15) applied Buser´s criteria, whereas Cordaro *et al*. (14) applied Albrektsson´s criteria. Success rates for delayed implants ranged from 96.9% to 100%, with a mean of follow-up period of 12-24 months; but it is noteworthy that the number of implants evaluated in these three studies represented over three quarters of the total number of implants placed in grafted jaws ( Table 2). Success rate for simultaneous implants was 89.5% at 12-month follow-up (15). The marginal peri-implant bone loss varied from 0.08±0.9 (16) to 0.20±0.50 mm (15) for delayed implants, and of 0.69±0.67mm for simultaneous implants (15). So, despite the small number of studies reviewed, it would appear that delayed implant placement may be preferable to simultaneous placement.

**Table 2:**
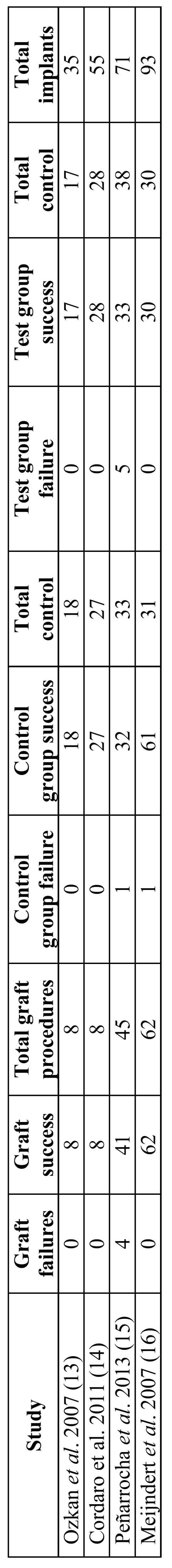
Descriptive results from studies with horizontal augmentation procedures.

- Vertical augmentation

Patient and intervention characteristics

Two studies included 54 patients with 120 dental implants. A total of 28 patients with 64 dental implants underwent vertical ridge augmentation with intraoral onlay block bone grafts, all with delayed placement (17,18) (see  Table 3 for further details).

**Table 3:**
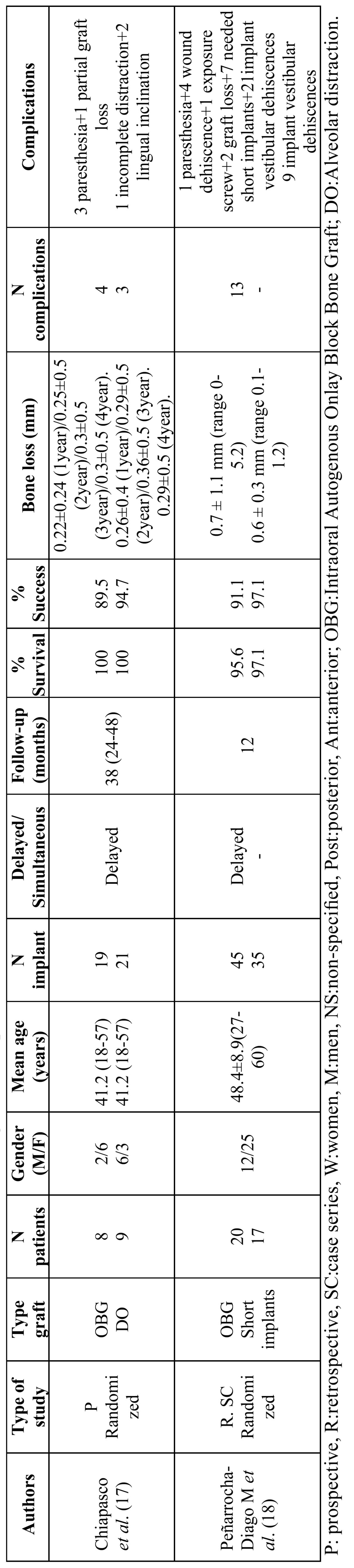
Studies included in the review on vertical augmentation procedures.

Outcomes

With regard to postoperative surgical complications, Chiapasco *et al*. (17) reported 3 paresthesias and one partial bone graft. Bone resorption before implant placement was significantly higher in the block bone graft group; although no significant differences as far as peri-implant bone resorption after implant placement were found. Peñarrocha *et al*. (18) reported 4 graft exposures, 2 graft losses, one screw head exposure and one paresthesia; it was also reported that the bone gain was not sufficient to place 10 mm-long dental implants in seven patients, so 7 mm implants were placed instead; and 21 dental implants needed additional particulate bone graft due to peri-implant dehiscences during implant placement. None article studied the soft tissues healing or esthetic outcomes. In both studies, implants were not placed until four to five months after bone grafting procedure.

Implant survival varied from 95.6% after one year post-loading (18) to 100% at a mean of 38 months post-loading (17). Implant success varied from 91.1% after one year post-loading (18) to 89.5% at a mean of 38 months post-loading (17). Chiapasco *et al*. (17) obtained an implant success rate of 85.9% for the graft group and 94.7% for the alveolar distraction group. For Peñarrocha *et al*. (18), the success rate was 91.1% for the graft group and 97.1% for the short implant group. Descriptive results suggested that there was a slightly lower success rate for implants placed with bone grafts than their respective control groups ( Table 4). Due to the heterogeneity of the data of these studies (different control groups) it was not possible to perform a metaanalysis. Peñarrocha-Diago *et al*. (18) obtained a peri-implant marginal bone loss for the bone grafting group of 0.7±1.1 mm at the twelve-month follow-up, while Chiapasco *et al*. (17) obtained 0.22±0.24 mm after twelve months, and 0.3±0.5 mm at a four-year follow-up (17). The difference in peri-implant marginal bone loss between these studies may be attributed to the fact that Chiapasco *et al*. (17) placed dental implants in a supracrestal position, while Peñarrocha-Diago *et al*. (18) submerged the smooth implant necks into the bone slightly, so that slight resorption could be expected at that level. Although Peñarrocha *et al*. (18) did not find statistically significant differences in survival or success between the groups, mean marginal bone loss was higher for implants placed in regenerated bone than for short implants; the authors proposed that when residual bone height over the mandibular canal is between 7 and 8 mm, short implants (with a 5.5 mm intrabony length) might be a preferable treatment option over vertical augmentation, reducing chair time, economic cost and morbidity.

**Table 4:**
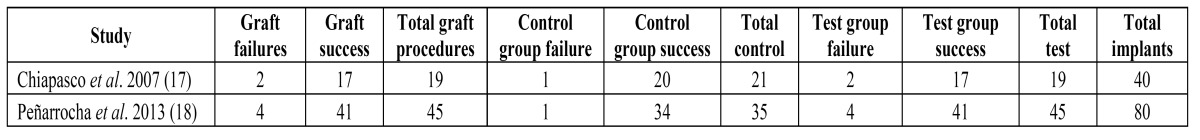
Descriptive results from studies with vertical augmentation procedures.

- Quality assessment of trials and risk of bias

One study (16) had a low risk of bias, while the other 5 (13-15,17,18) presented a high risk of bias.

## Discussion

The data reported in the literature seem to demonstrate that bone augmentation by means of the positioning of intraoral onlay grafts can be considered a reliable surgical technique for obtaining sufficient bone volume for the placement of dental implants where it would not otherwise be possible (4). However, the capacity of bone grafts to restore original bone volume varies, and the results reported in the literature are contradictory due to differences in observation periods, type and site of reconstruction, timing of implant loading, and last but not least, the site of bone harvesting (1). Since geometry of the defects and residual bone differ in horizontal or vertical bone atrophy, studies were group accordingly separately.

Common techniques introduced for horizontal bone augmentation are guided bone regeneration, ridge splitting and expansion, and block grafts (2). In a systematic review by Fiorellini and Nevins (7) guided bone regeneration showed an implant survival rate of 95.8% ± 5.3% in 56.5 ± 25.5 months. Other reviews (1,3-6) found that implant survival rates were 95.5% for guided bone regeneration, 90.4% for onlay grafts, and 91% to 97.3% for ridge expansion, while ridge augmentation success rates were 60% to 100% for guided bone regeneration, 92% to 100% for on-lay block grafts, and 98% to 100% for ridge expansion. The main criteria to consider the timing of implants are the residual bone volume needed to allow a correct implant position and angulation, and the bone density needed to achieve primary implant stability (1). In the present systematic review, survival rates ranged from 96.9% to 100%. No differences were found in the survival rates of implants placed with block bone grafts or guided bone regeneration, and these outcomes were similar to implants placed in native bone.

More challenging is the vertical augmentation procedure; a variety of surgical procedures have been proposed, such as: autogenous bone grafts, vertical guided bone regeneration, and alveolar distraction osteogenesis. As many atrophic alveolar ridges are deficient in height and width, they may require flattening for better graft adaptation (19). A second concern is the adequate adaptation of the bone graft, which is critical for graft success (19). However, despite a relevant number of publications reporting favorable results with these three different surgical procedures, considerable controversy still exists as far as the choice of the more reliable technique is concerned, due to the lack of comparative studies (1). In order to be more conservative and reducing the morbidity, the use of short implants in cases of reduced bone height in the posterior mandible has been encouraged in recent systematic reviews (20,21). In the present review, only two studies fulfilled the inclusion criteria and control groups were different so a metaanalysis was not performed. However, the results from both studies suggested a slightly lower success rate for implants placed with bone grafts than their respective control groups (distraction osteogenesis and short dental implants).

Among the disadvantages of lateral or vertical bone grafting procedures is the resorption of a significant proportion of the graft (14,22-24). Antoun *et al*. (22) found a significant difference in resorption width, with a mean of 0.3 mm in the membrane group versus 2.3 mm in the graft group without membrane use. Because the use of non-re sorbable membranes involves the more complex clinical handling of the soft tissues, some authors proposed the use of re sorbable collagen membranes and anorganic bovine bone to protect the block graft and prevent its resorption (23). Maiorana *et al*. (24) evaluated the effect of bovine bone by comparing block grafts alone versus block grafts protected by bovine bone particles; the authors obtained 18.3% graft block resorption compared with 9.3% resorption when grafts were covered with the bone substitute. Cordaro *et al*. (14) added a second resorb able membrane, finding minimal graft resorption during healing (0.25 mm - 5.5% of the whole graft); meanwhile, significantly greater graft resorption was observed (0.89 mm - 21% of the whole graft) in the graft alone group. In any case, oversized grafts should be harvested in order to maintain sufficient graft volume after the initial resorption phase (1). Regarding vertical augmentations, there is an increased risk of graft exposure because when expanding the soft tissue envelope vertically, the forces exerted on the graft may lead to major resorption (10,25). Rocuzzo *et al*. (10) reported that sites with Ti-Mesh coverage suffered bone resorption of 13.5% and Proussaefs & Lozada (25) presented an average of 17.4% resorption after 4-6 months. In both cases, resorption rates were higher than in lateral augmentations. Therefore, the assessment of esthetic parameters and soft tissues stability should be encouraged in order to identify long-term complications on hard and soft peri-implant tissues. Cordaro *et al*. (14) and Chiapasco *et al*. (17) studied several clinical parameters (Modified plaque index, modified bleeding index and probing depth) but they did not collect the presence of peri-implant recessions, mucositis or esthetic issues. Hiatt and Schallhorn (26) found that the degree of regeneration directly correlates to the adequacy of soft tissue cover and the surface area of the vascularized defect bony walls, implying that primary wound coverage is imperative for bone regeneration. Therefore, a minimum thickness of 1.5 mm is advocated to provide additional protection and coverage of the augmented bone site. According to Tolman (27) and Brener (28) wound dehiscence was directly related to implant failure. Temporary mental paresthesia after harvesting chin grafts ranges from 10% to 50%, whereas the mandibular ramus ranges from 0% to 5% (29,30).

The main limit encountered in this literature review was the lack of studies with a comparative design and randomized controlled studies. Future research must include control groups and standardized criteria for defining implant success or failure for both simultaneous and delayed protocols, in order to obtain rigorous evidence-based results. In this way, the data presented in this review should be considered indicative rather than conclusive.

## Conclusions

Survival and success rates of implants placed in horizontally and vertically resorbed edentulous ridges reconstructed with block bone grafts are similar to those of implants placed in native bone, in distracted sites or with guided bone regeneration. More surgical challenges and morbidity arise from vertical augmentations, thus short implants may be a feasible option. Our recommendations for future research focus on the performance of large-scale randomized controlled studies with longer follow-ups involving the assessment of esthetic parameters and hard and soft peri-implant tissue stability.
